# Early-life tobacco exposure is causally implicated in aberrant RAG-mediated recombination in childhood acute lymphoblastic leukemia

**DOI:** 10.21203/rs.3.rs-4510345/v1

**Published:** 2024-06-20

**Authors:** Adam de Smith, Tanxin Liu, Keren Xu, Anmol Pardeshi, Swe Swe Myint, Alice Kang, Libby Morimoto, Michael Lieber, Joseph Wiemels, Scott Kogan, Catherine Metayer

**Affiliations:** University of Southern California; University of Southern California; University of Southern California; University of Southern California; Laboratory of Cancer Epigenome, Division of Medical Sciences, National Cancer Centre Singapore, Singapore; University of California Berkeley; UC Berkelely; Norris Comprehensive Cancer Center, University of Southern California Keck School of Medicine; USC Keck School of Medicine; University of California San Francisco; University of California

## Abstract

Acute lymphoblastic leukemia (ALL) is the most common cancer in children, yet few environmental risk factors have been identified. We previously found an association between early-life tobacco smoke exposure and frequency of somatic deletions of 8 leukemia driver genes among childhood ALL patients in the California Childhood Leukemia Study. To expand analysis genome-wide and examine potential mechanisms, we conducted tumor whole-genome sequencing in 35 ALL patients, including 18 with high prenatal tobacco exposure and 17 with low exposure as determined by established epigenetic biomarkers. High tobacco exposure patients had significantly more structural variants (P < .001) and deletions (P = .001) genome-wide than low exposure patients. Investigation of off-target RAG recombination revealed that 41% of deletions in the high tobacco exposure patients were putatively RAG-mediated (full RAG motif identified at one or both breakpoints) compared with only 21% in the low exposure group (P = .001). In a multilevel model, deletions in high tobacco exposure patients were 2.44-fold (95% CI:1.13–5.38) more likely to be putatively RAG-mediated than deletions in low exposure patients. No point mutational signatures were associated with prenatal tobacco exposure. Our findings suggest that early-life tobacco smoke exposure may promote leukemogenesis by driving development of somatic deletions in pre-leukemic lymphocytes via off-target RAG recombination.

## Introduction

Acute lymphoblastic leukemia (ALL), the most common malignancy of childhood, is characterized by uncontrolled clonal expansion of abnormal, immature B- or T-lymphoblasts and their progenitors through sequential accumulation of cancer-driving mutations [1]. A “two-hit” model of leukemogenesis was proposed to delineate the natural history of ALL. In most pediatric ALL cases, pre-leukemic clones are initiated during fetal development through the formation of chromosomal abnormalities, such as *ETV6::RUNX1* fusion or high hyperdiploidy [2]. The genetic lesions that develop in utero are not sufficient to cause ALL, but instead generate a clinically silent pre-leukemic phase [3]. During early childhood, a small fraction of covert pre-leukemic clones acquire postnatal “second hit” mutations and copy-number alterations and progress towards overt leukemia [2]. Common second hit alterations in ALL include deletions of genes regulating cell cycle control (*CDKN2A* and *RB1*) and B-lymphocyte development and differentiation (*PAX5, IKZF1, ETV6*, and *EBF1*) [4, 5].

A multifactorial mix of exposure and inherited genetic background could play a role in the two-hit model of leukemogenesis. Studying environmental exposures in childhood cancer etiology poses several challenges, including a lack of large prospective cohort studies due to low incidence rates, a reliance on retrospective case-control studies and potentially inaccurate exposure assessment based on parent interview data, ignorance of the critical windows of exposure, and difficulties in exposure measurements during pre- and postnatal development. Investigating the association between environmental exposures and mutational patterns and signatures in ALL tumor sequencing data represents an alternative and complementary approach to traditional cancer epidemiological studies for studying risk factors [6].

Previous studies have shown that the formation of deletions through “off-target” V(D)J recombination is a mechanism that drives the development of overt ALL [7–9]. The recombination-activating gene (RAG) proteins, encoded by RAG1 and RAG2, help to generate antibody diversity by inducing DNA double strand breaks and recombining the variable (V), diversity (D) and joining (J) gene segments, allowing the generation of diverse immunoglobulin and T-cell receptors during the early stage of B cell and T cell maturation [2, 10]. During the maturation of B cells, the activation-induced cytidine deaminase (AID) enzyme initiates the class switch recombination (CSR) and somatic hypermutation (SHM) processes to switch the class of the antibody and introduce random mutations in the variable region, contributing to a diverse repertoire of antibodies tailored to varying antigen specificities. The same AID-dependent modification processes are also involved in illegitimate recombination at a broad range of oncogenes [11–14], and can induce secondary copy-number alterations in childhood ALL [1, 2].

Little is known regarding exogenous factors that may drive development of somatic alterations in childhood ALL, although it has been suggested that childhood exposure to infections may trigger progression to overt ALL [2]. In addition, we previously found that the frequency of somatic deletions of driver genes in childhood ALL patients is positively associated with early-life tobacco smoke exposure. Our finding was confirmed using both parental reports of tobacco smoking during pregnancy as well as established epigenetic biomarkers of prenatal tobacco smoke exposure [15, 16]. Our prior studies were limited to the copy-number analysis of eight genes commonly deleted in ALL and targeted by a multiplex ligation-dependent probe amplification assay, which did not provide information on deletion breakpoint sequences [16]. Previous studies demonstrating that increased ‘illegitimate’ genomic deletions mediated by V(D)J recombinase was associated with passive maternal tobacco exposure [17] as well as hematologic malignancies [7] motivated us to investigate whether tobacco smoke exposure during pregnancy may be associated with off-target RAG recombination-mediated deletions in childhood ALL. In this study, we performed tumor whole genome sequencing (WGS) in childhood ALL patients with either high prenatal tobacco smoke exposure or low exposure, to explore the hypothesis that tobacco smoke exposure may lead to the development of secondary somatic alterations that arise via RAG recombination mechanisms.

## Methods

### Study subjects

This study was reviewed and approved by the Institutional Review Boards at the University of Southern California, the University of California, Berkeley, the California Department of Public Health, and all participating hospitals. Written informed consent was obtained from all study participants. This study was conducted in accordance with the Declaration of Helsinki.

Childhood ALL patients were included from the California Childhood Leukemia Study (CCLS), a case-control study conducted from 1995 to 2015 to identify genetic and environmental risk factors for childhood leukemia, and described in **Supplementary Methods** and in detail elsewhere [18]. For the current study, we selected two groups of ALL patients that we categorized as having “high” (N = 18) or “low” (N = 17) early-life tobacco exposure based on established epigenetic biomarkers (**Figure S1**) [15, 16, 19], as described in **Supplementary Methods**.

### Whole-genome sequencing

WGS was performed for the 35 matched tumor-normal pairs. Further details including quality control assessment of sequencing data, methods for detecting somatic variants, including single nucleotide variants (SNVs), indels, and structural variants (SVs), and mutational signature analyses are described in **Supplementary Methods**.

### Deletion breakpoint motif analysis

We obtained +/− 50bp flanking sequences from each deletion breakpoint based on hg38 coordinates. Recombination signal sequence (RSS) motif enrichment analysis was performed using the Find Individual Motif Occurrences (FIMO) tool in MEME suite v5.5.5 (*P* < 10^− 4^) [20, 21]. In brief, FIMO searches a set of individual sequences for the occurrence of known motifs provided by the user, treating each motif independently [20]. The position-weight matrix (PWM) used to identify RSS motifs were obtained from previous studies [7, 22, 23], assuming a background rate of 0.2 for C/G and 0.3 for A/T. We investigated the presence of the full RSS motif, which can include a 12- or 23-nucleotide spacer, and heptamer and nonamer motifs within 50 bp flanking each deletion breakpoint. This was conducted initially for deletions in both immunoglobulin/T-cell receptor (Ig/TCR) and non-Ig/TCR regions, and subsequently limited to non-Ig/TCR regions (where both breakpoints were outside +/− 1000 bp of Ig/TCR regions) to examine off-target RAG recombination. The coordinates of “on-target” Ig/TCR (IgH, IgK, IgL, TRB, TRA/TRD, TRG) regions were based on prior studies [5, 24] (**Table S1**). To explore the distance and clustering of motifs, we identified the motif signal decay within 5–200 bp from deletion breakpoints and plotted the proportion of deletions with at least one RSS motif.

*De novo* deletion breakpoint motif analysis was conducted using HOMER v.4.11 [25]. We selected +/− 50bp from the deletion breakpoints and used repeat masked sequences. We first searched for motifs ranging from 5 to 12 bp and then specified the length of motifs to be 7 bp (heptamer). We did not investigate the full RSS motif using HOMER as the recommended maximum motif length was 15 bp.

Analysis of non-templated nucleotides (NTN) inserted at deletion breakpoints, a hallmark of RAG recombination, is described in **Supplementary Methods**.

### Statistical analyses

Details on the statistical methods used are included in **Supplementary Methods**. All p-values were two-sided, and p-values < 0.05 were considered statistically significant. Analyses were performed using *R*.

## Results

Tumor-normal WGS was performed for 35 childhood ALL patients, including 18 with high prenatal tobacco smoke exposure and 17 with low exposure based on epigenetic biomarkers. The majority of patients (31/35) were of the B-cell immunophenotype, with 43% Hispanic/Latino, 37% non-Hispanic white, and 20% non-Hispanic other based on self-reported race/ethnicity (**Table 1, Table S2**).

**Figure 1:**
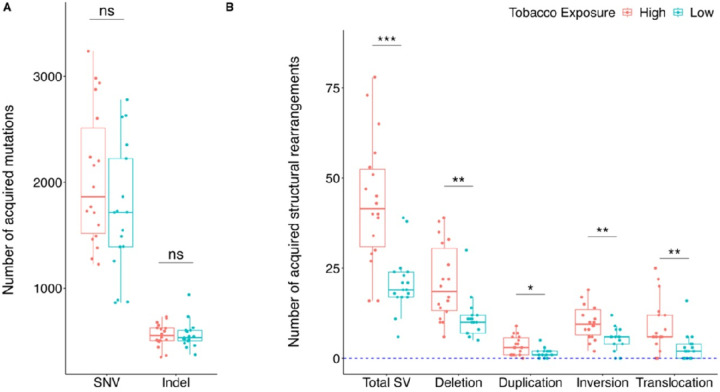
Number of genome-wide somatic alterations by prenatal tobacco exposure status. Single nucleotide variants (SNVs), insertion deletion polymorphisms (indels), and structural variants (SVs: deletions, duplications, inversions, translocations) were called from whole-genome sequencing data in 35 paired tumor-normal samples. The number of different somatic alteration types in the high tobacco exposure (n=18) and low tobacco exposure (n=17) childhood ALL patients is displayed by box and whisker plots. Statistical comparisons were performed using Wilcoxon rank sum tests. *** P<.001; ** P<.01; * P<.05

We obtained an average sequencing read depth of ~ 35X for germline and 58X for tumor samples and identified a total of 66,190 SNVs and 19,111 indels with VAF > 0.10, including 2503 (2.9%) SNV/indels in coding regions. On average, patient tumor samples harbored 1891 SNVs (median: 1729; range:864–3238; IQR: 868) and 546 indels (median: 535; range:350–732; IQR: 109) (mean coding SNVs/indels per patient: 58; range: 16–250). The known ALL driver genes most commonly affected by SNV/indels were *KRAS, FLT3, JAK2, PAX5, ERG, PTPN1, NF1* and *RB1* (**Figure S2**). We did not find statistically significant differences in the number of SNVs (*p* = 0.198) or indels (*p* = 0.843) between the high tobacco exposure and low exposure ALL patients (Fig. 1).

**Figure 2:**
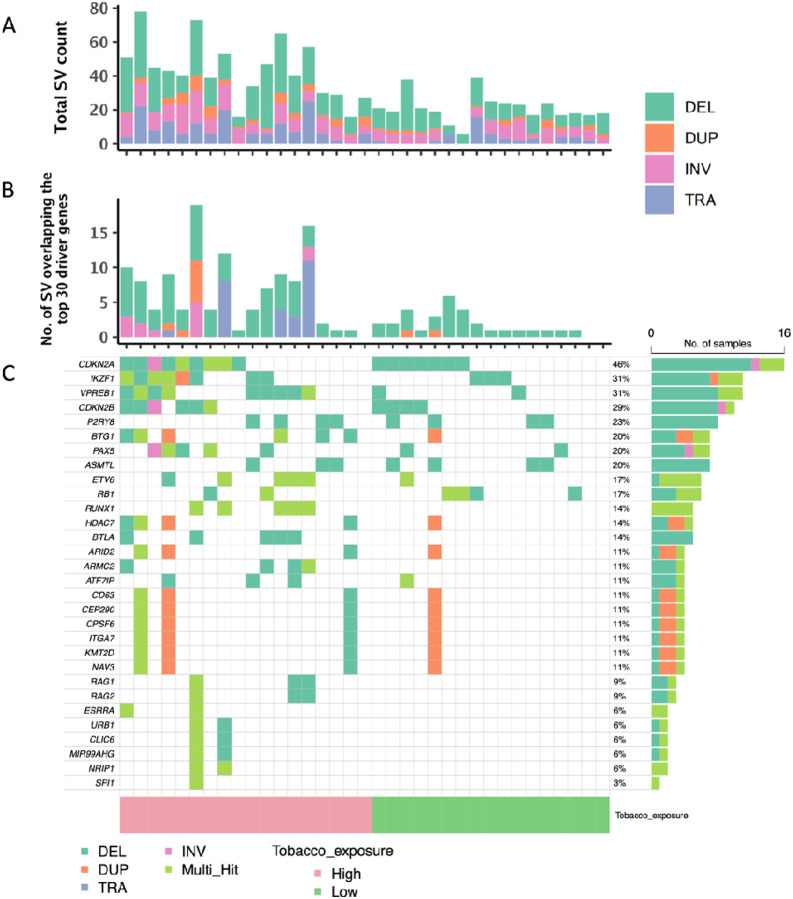
Somatic structural variants and their overlap with childhood ALL driver genes. The total number of structural variants (SVs) per patient (A) was based on the overlap of calls by three SV detection tools (Lumpy, Manta, and Delly), with final numbers of SVs based on those detected by at least two out of three methods. Total SV count was based on the sum of counts of different SV types: deletions (DEL), duplications (DUP), translocations (TRA), and inversions (INV). The middle plot (B) displays the number of SVs and different SV types per patient that overlapped known childhood ALL driver genes, limited to the top 30 affected genes in our dataset. The bottom oncoplot (C) shows the ALL driver genes affected by SVs across the 35 patients, with 32 (91.4%) out of 35 patients harboring at least one SV overlapping an ALL driver gene, and genes ordered by the number of affected patients. SVs annotated as “Multi_Hit” indicate where the same gene was affected by more than one SV in the same patient. For all three plots, childhood ALL patients were stratified by tobacco exposure status as indicated.

We identified a total of 1140 SVs (566 deletions, 90 duplications, 273 inversions and 211 translocations) with VAF > 0.10 among the 35 ALL patients, with a median of 33 total SVs (range 6–78; IQR: 23), 12 deletions (range 5–39; IQR: 11), 2 duplications (0–9; 2.5), 6 inversions (0–19; 4.5), and 4 translocations (0–25; 4.5) per patient. Of these, 282 SVs overlapped known ALL driver genes [5, 26–28], with a median of 6 ALL driver gene SVs (range 1–23; IQR:10) per patient. Eighty-five percent of SVs were < 1Mb in size (11% were 1Mb to 10Mb, and 4% were > 10Mb). Figure 2 displays the known ALL driver genes most frequently affected by SVs, including *CDKN2A (46%), IKZF1 (31%), VPREB1 (31%), CDKN2B (29%), P2RY8 (23%), BTG1 (20%), PAX5 (20%), ASMTL (20%), ETV6 (17%), RB1(17%), RUNX1 (14%), HDAC7 (14%)* and *BTLA* (14%).

A significantly higher number of genome-wide total SVs (*p* < 0.001), deletions (*p* = 0.001), duplications (*p* = 0.017), inversions (*p* = 0.002), and translocations (*p* = 0.004) was found in the high tobacco exposure patients compared with the low exposure group (Fig. 1; **Table 1**). When limiting to SVs overlapping known ALL driver genes, total SVs and deletions remained significantly increased in the high exposure group (**Table S3, Figure S3**). Age-at-diagnosis was positively associated with the number of SVs (*p* = 0.009) and the number of overall deletions (*p* = 0.0005) (**Table S4**).

### Analysis of RAG sequences at deletion breakpoints

To explore the hypothesis that tobacco exposure-related gene deletions in childhood ALL bear the hallmark of RAG recombination activity, we first searched deletion breakpoint sequences for the occurrence of RAG motifs using FIMO [20, 21]. We considered presence of the full RSS motif at one or both deletion breakpoints as good evidence of RAG recombination, and presence of only the heptamer or nonamer motif as weaker evidence. Among 566 deletions in total, 255 (45.1%) had at least one breakpoint located in Ig/TCR regions (on-target), and 311 (54.9%) deletions had both breakpoints in non-Ig/TCR regions and may therefore be mediated by off-target RAG recombination. Ninety-three percent of Ig/TCR deletions and 35% of non-Ig/TCR region deletions had a full RSS motif within 50 bp of at least one of the two breakpoints (*i.e*., putatively RAG-mediated). The high tobacco exposure ALL patients had a higher total number of putatively RAG-mediated deletions compared with the low tobacco exposure group overall (*p* = 0.002), and when limited to non-Ig/TCR deletions (*p* = 0.003) or Ig/TCR deletions (*p* = 0.005). High tobacco exposure was also associated with a significantly higher number of deletions with a full RSS motif at both breakpoints in non-Ig/TCR regions (*p* = 0.004) but not in Ig/TCR regions (*p* = 0.472) (**Table S5**).

**Figure 3:**
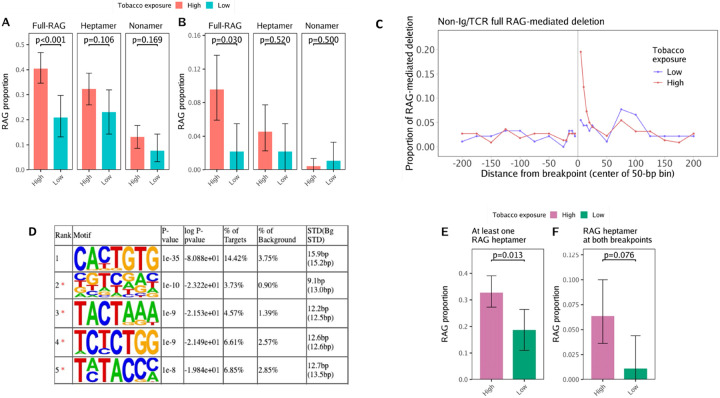
Off-target RAG-mediated deletions by tobacco exposure status. Bar plots displaying the proportion of deletions in non-Ig/TCR (*i.e.*, off-target) regions with at least one breakpoint (**A**) or with both breakpoints (**B**) having an RSS motif detected by FIMO, in childhood ALL patients with high tobacco exposure (n=18) or low tobacco exposure (n=17). A total of 220 non-Ig/TCR region deletions were detected in high tobacco exposure patients and 91 non-Ig/TCR deletions in low exposure patients. Error bars represent 95% bootstrapped confidence intervals. Chi-square tests were used to compare the proportions of deletions with at least one RAG motif (either full, heptamer or nonamer) between two groups. Fisher’s exact tests were used to compare the proportions of deletions with RAG motif (either full, heptamer or nonamer) at both breakpoints between two groups. (**C**) The proportion of non-Ig/TCR (*i.e.*, off-target) putatively RAG-mediated deletions with at least one full RSS motif was plotted against the distance of the motif from the deletion breakpoint, ranging from within 5-bp to 200-bp. A positive distance represents bases interior to the deletion breakpoint (inside the deletion) and a negative value represents bases exterior to the breakpoint (outside the deletion). Proportions are displayed for 35 childhood ALL patients overall, and for the high tobacco exposure (n=18) and low exposure (n=17) patients separately. The table (**D**) displays results from HOMER de novo motif discovery analysis for motifs with length=7bp found in sequences +/− 50 bp from each deletion breakpoint, limited to non-Ig/TCR (off-target) deletions. The top 5 most significant motifs are displayed. The most significant motif “CACTGTG” corresponds to the RAG heptamer. Motifs labeled with asterisks were possible false positive results according to HOMER. Bar plots below show the proportion of deletions with at least one breakpoint (*p* value from Chi-square test) (**E**) or with both breakpoints (*p* value from Fisher’s exact test) (**F**) at which the RAG heptamer was identified by HOMER, in childhood ALL patients with high tobacco exposure (n=18) or low tobacco exposure (n=17).

Given that the number of putatively RAG-mediated deletions in the high tobacco exposure versus low exposure patients may simply reflect the frequency of overall deletions in each patient group, we next examined the *proportion* of deletions that appeared to be mediated by off-target RAG recombination. In Ig/TCR regions, we did not find a significant difference in the proportion of putatively RAG-mediated deletions between high and low tobacco exposure groups but did find that low tobacco exposure patients had a significantly higher proportion of Ig/TCR deletions with RAG motif at both breakpoints (p = 0.019) (**Figure S4**). Conversely, in non-Ig/TCR regions, high tobacco exposure patients harbored a significantly higher proportion of putatively RAG-mediated deletions than in the low exposure group (40.5% vs. 20.9%; *p* = 0.001, Chi-square test) (Fig. 3, **Table S6, Figure S5-S6**). High tobacco exposure patients also had a higher proportion of non-Ig/TCR deletions with at least one RAG heptamer (32.3% vs 23.1%) and deletions with at least one RAG nonamer (13.2% vs 7.7%) at the breakpoints, albeit not statistically significant. We also identified a higher proportion of non-Ig/TCR deletions with the full RSS motif at both breakpoints in the high exposure group compared to the low exposure group (9.5% vs 2.2%; *p* = 0.0297, Fisher’s exact test) (Fig. 3). This off-target effect was in the opposite direction of the on-target effects observed in Ig/TCR regions, suggesting that there may be a skewing towards off-target RAG recombination in the high tobacco exposure group. In support that these are true RAG-mediated events, the RSS motif (full RSS, heptamer or nonamer) was largely internal to the breakpoint of deletions in both high and low tobacco exposure groups (Fig. 3C, **Figure S7A-B**). Further, 121/124 (97.6%) of heptamers located internal to the breakpoints of non-Ig/TCR deletions and 394/398 (99.0%) of heptamers internal to the breakpoints of Ig/TCR deletions were found in the correct orientation for typical V(D)J recombination where the RAG motifs are deleted in the form of “excision circles”.

We also performed agnostic *de novo* motif analysis using HOMER, which identified the RAG heptamer (14.42% of targets; *p* = 1e-35) as significantly enriched within 50 bp of the deletion breakpoint junctions (Fig. 3D). Consistent with FIMO results, high tobacco exposure patients harbored a higher proportion of deletions with at least one off-target RAG heptamer (high vs. low groups: 32.7% vs 18.7%; p = 0.013) and a higher proportion of deletions with off-target RAG heptamer at both breakpoints (6.4% vs 1.1%; *p* = 0.076, Fisher’s exact test) (Fig. 3E–F, **Table S7**). We investigated additional significant motifs identified by HOMER but found no motifs with a target frequency above 5% (**Figure S8**).

Age-at-diagnosis was positively associated with the number of non-Ig/TCR putatively RAG-mediated deletions (*p* = 0.0008) as well as the proportion of non-Ig/TCR RAG-mediated deletions by patient (*p* = 0.005) (**Table S4**). In a multilevel model, non-Ig/TCR deletions identified among the high prenatal tobacco exposure patients had 2.44-fold higher odds (95%CI: 1.13, 5.38) of being putatively RAG-mediated than deletions in the low exposure group (**Table 2**). We found an even stronger association in the multilevel model between high tobacco exposure and deletions in which the full RSS motif was found at both breakpoints (OR, 4.70, 95%CI: 1.34, 29.75) (**Table 2**). Further analyses and statistical modeling of putatively RAG-mediated deletions, including in relation to age-at-diagnosis, ethnicity, and additional features, are presented in the **Supplementary Information** and **Table S8**.

To provide support for RAG recombination that we assigned from our motif analysis, we investigated the presence of NTN sequences inserted at deletion breakpoints. Among 311 non-Ig/TCR deletions, 231 (74.3%) had NTN at deletion breakpoints. Deletions with the RAG motif at both breakpoints had the highest proportion of NTN (92.31%), followed by deletions with only one breakpoint with a RAG motif (79.3%) and then non-RAG-mediated deletions (64.53%). Manual inspection of 20 non-Ig/TCR deletions with RAG motif enrichment at both breakpoints using IGV revealed that 19 (95%) deletions had NTN, similar to the number (n = 18, 90%) identified by the overlapped segment between the upstream and downstream deleted sequences.

### Mutational signature analysis

In the *de novo* signature analysis using SigProfilerExtractor, four *de novo* SBS were considered as the best solution and were decomposed into seven COSMIC signatures: SBS1 and SBS5 (clock-like), SBS2 and SBS13 (AID/APOBEC activity), SBS3 (defective homologous recombination-based DNA damage repair), SBS18 (possibly damage by reactive oxygen species (ROS)), and SBS30 (deficiency in base excision repair due to inactivating mutations in *NTHL1*) (**Table S9–10, Figure S9-S10**). The AID/APOBEC signature was identified in only two of the 35 patients, both of which were in the high-tobacco exposure group. For signatures detected in both tobacco exposure groups (clock-like and ROS), we did not observe any significant difference in either total number of signature-related mutations between exposure groups or proportion of signature-related mutations in each patient between the two groups (*p* > 0.05) (**Table S10–11, Figure S10**). No significant difference was found for the proportion of subjects carrying each of the signatures between the two groups (**Table S12**). Age-at-diagnosis was positively associated with the number of SBS1-related mutations (linear regression beta:30.89, *p* = 0.0003). Results were similar for analysis of SBS mutational signatures using SigProfilerAssignment and DeconstructSigs (**Supplementary Information, Table S13–14, Figure S11–13**). Similar to SBS results, we found no significant difference between high and low tobacco exposure patients for any indel or double-base substitution signatures (**Table S9**).

## Discussion

In this study, we have extended upon our prior investigations into the association between prenatal tobacco exposure and somatic copy-number deletions in pediatric ALL patients, by examining structural variation genome-wide and investigating potential mutational mechanisms. Our findings support that the relationship between early-life tobacco smoke exposure and frequency of deletions in ALL patients may be mediated by its effects on the developing immune system, specifically resulting in increased off-target RAG recombination, and highlighting the potential mutagenic effects of a preventable environmental exposure.

In previous analyses, we found that early-life tobacco exposure was associated with an increased frequency of deletions at eight regions commonly lost in pediatric ALL tumors, including *CDKN2A, ETV6, IKZF1, PAX5, RB1, BTG1, EBF1*, and *CRLF2-P2RY8* region [16]. Our current study focused on a subset of patients examined previously, with high or low early-life tobacco exposure based on established epigenetic biomarkers. Rather than a true replication, we expanded our analysis genome-wide and considered other types of alterations. In addition to the previously studied genes, we found relatively high frequencies of deletions impacting other known ALL driver genes, in particular at *VPREB1* but also including *ASMTL, BTLA,* and *ATF7IP*. Moreover, in addition to finding an association between tobacco smoke exposure and somatic deletions genome-wide, we found a significantly higher frequency of translocations, inversions, and overall SVs in the high versus low tobacco exposure patients, suggesting that early-life tobacco smoke exposure may be associated with general genomic instability in ALL tumor samples.

Case-control studies of parental tobacco smoking based on questionnaire data, which can be subject to misclassification bias, have shown inconsistent associations with pediatric ALL risk [29–31]. We recently reported a lack of association between DNA methylation at the *AHRR* CpG cg05575921, an epigenetic biomarker of maternal smoking during pregnancy, and childhood ALL risk in a case-control study of ~ 3,000 ALL cases and ~ 3,200 controls [32], which supported the previous epidemiological literature regarding all childhood ALL combined [33]. Although results from our case-only analyses may appear inconsistent with case-control study findings, they suggest that tobacco smoke exposure may have tumor subtype-specific effects on ALL development.

Tumor WGS enabled us to investigate potential mutational mechanisms by examining breakpoint sequences. In particular, we focused on off-target RAG recombination given that this has both been implicated in the formation of somatic alterations in childhood ALL and associated with prenatal tobacco smoke exposure in cord blood lymphocytes [7–9, 17]. The significantly higher frequency of RAG-mediated deletions in high tobacco exposure patients compared with low exposure patients was unremarkable, as this may simply have correlated with the overall higher number of deletions in the former. However, when examining the proportion of deletions that appeared to be mediated by off-target RAG recombination, the significant positive association with early-life tobacco exposure supports a potential leukemogenic role in childhood ALL development.

V(D)J recombination activating genes *RAG1* and *RAG2* have been implicated in the formation of gene deletions in some ALL subtypes, including *ETV6::RUNX1* ALL and T-cell ALL [7, 8, 34]. Our findings bear similarities to a previously reported genome-wide analysis of structural rearrangements in *ETV6::RUNX1* pediatric B-ALL [7], indicating that a preponderance of deletions resulted from off-target RAG recombination. There is a paucity of evidence on the potential influence of environmental exposures on RAG recombination activity, although two prior studies have demonstrated that maternal secondhand exposure to cigarette smoke was associated with a significant increase in deletions of the *HPRT* reporter gene via illegitimate V(D)J recombination in cord blood T-lymphocytes [17, 35]. A recent study among current smokers showed that tobacco smoking influences long-persistent adaptive immune responses as well as increased inflammatory responses following bacterial stimulation [36]. Hence, the effects of tobacco smoke exposure in childhood ALL may not necessarily be directly mutagenic but have effects on the immune system, for example through upregulation of RAG proteins or via stalling of lymphocyte development given that RAG proteins are most active in immature B- and T-cells [37, 38]. Future studies could advance the understanding of inflammatory mechanisms of tobacco-related (epi) genetic changes and uncover the role of tobacco exposure in leukemogenesis.

Intriguingly, we found a positive association between childhood ALL patient age-at-diagnosis and the proportion of non-Ig/TCR RAG-mediated deletions in their leukemias. When including age-at-diagnosis in the multilevel model, we still observed a positive association between tobacco exposure and RAG-mediated deletions although the effect was attenuated. Age-at-diagnosis likely correlates with exposure as even women who are motivated to quit smoking during pregnancy rarely reach abstinence, and among those who do, most restart within a year of birth [39]; therefore, patient age may be a proxy for cumulative dose. It is also possible that age-at-diagnosis is related to differences in ALL molecular subtypes that in turn are associated with variation in the contribution of RAG recombination mechanisms and deletions. Indeed, presence of somatic deletions have previously been associated with older age-at-diagnosis in childhood ALL patients [40, 41]. Larger sample sizes will be required to deconstruct independent impacts of molecular subtypes and cumulative tobacco exposure on the observed increased rate of deletions with age-at-diagnosis in ALL patients.

To our knowledge, this is the first study to uncover a relationship between early-life tobacco exposure and off-target RAG-mediated deletions in childhood ALL. There were several strengths to our study. Tumor WGS enabled us to investigate structural variation at the genome-wide level. The use of epigenetic biomarkers of early-life tobacco exposure to identify ALL patients with high or low exposure would not be affected by recall bias. In support of the accuracy of our analysis strategy to identify RAG-mediated deletions, we found that the vast majority of RSS motifs were located internal to deletion breakpoints and in the correct orientation for typical V(D)J recombination, and observed a rapid decay in the enrichment of the full RSS motif with increasing distance from the breakpoints. We also confirmed the presence of RSS motifs at the breakpoints of ~ 93% of the 255 deletions located in Ig/TCR regions. Furthermore, the frequency of NTN sequences that we identified at deletion breakpoints among likely RAG- and non-RAG-mediated deletions – 82.4% and 64.5%, respectively – was remarkably similar to those previously reported in ALL patients by Papaemmanuil et al., in which NTN sequences were found at 84.0% of RAG-mediated deletions with resolved breakpoints but only 65.3% of non-RAG-mediated deletions [7]. In addition, the multilevel model, which accounted for the varying number of deletions in each patient, yielded similar significant associations to simply comparing the proportion of RAG-mediated deletions found overall in the two patient groups.

We are fully mindful of the limitations of our study. First, the small sample size of patients limited our ability to adjust for potential confounding factors, such as molecular subtype, that may impact the association between tobacco exposure and proportion of RAG-mediated deletions. Second, we had limited statistical power to detect differences in mutational signatures between the two patient groups. Third, we classified patients into two tobacco exposure groups based on epigenetic biomarkers, which may overlook variations in dosage and frequency of prenatal maternal smoking exposure and limit our ability to discern subtle associations or dose-response relationships. Fourth, regarding tobacco exposure assessment, we used DNA methylation array data derived from newborn dry blood spots, and thus we could not consider postnatal tobacco exposures in the selection of patients or in our analyses. It is possible that epigenetic biomarkers of prenatal tobacco exposure in newborns may be correlated with postnatal exposure to parental smoking during childhood, which is a more relevant time window of exposure given that second hit deletions in ALL are thought to arise postnatally [42, 43]. We also cannot rule out that another unmeasured environmental exposure that is correlated with prenatal tobacco exposure may be the causal factor that drives RAG-mediated deletion formation. Further studies are needed to confirm our findings and to understand the precise biological mechanisms and the timing of exposures that underlie the association between tobacco exposure and deletions in ALL tumors, to inform future preventive strategies.

## Data Availability

This study used biospecimens from the California Biobank Program. Any uploading of genomic data and/or sharing of these biospecimens or individual data derived from these biospecimens has been determined to violate the statutory scheme of the California Health and Safety Code Sects. 124980(j), 124991(b), (g), (h), and 103850 (a) and (d), which protect the confidential nature of biospecimens and individual data derived from biospecimens. Should we be contacted regarding individual-level data contributing to the findings reported in this study, inquiries will be directed to the California Department of Public Health Institutional Review Board to establish an approved protocol to utilize the data, which cannot otherwise be shared peer-to-peer. Full results for somatic mutations and structural variants identified in each tumor sample by whole-genome sequencing and results of RSS motif analysis are included in the **Supplementary Data** files.
